# Editorial on Special Issue: “Advances in Nanotechnology-Based Drug Delivery Systems”

**DOI:** 10.3390/pharmaceutics17081038

**Published:** 2025-08-10

**Authors:** Carla Serri

**Affiliations:** Department of Medicine, Surgery and Pharmacy, University of Sassari, via Muroni 23/A, 07100 Sassari, Italy; cserri@uniss.it

## Abstract

Nanotechnology enables the design and application of nanostructures to improve drug delivery by modulating release, enhancing solubility, and increasing bioavailability of poorly soluble APIs, while reducing side effects. This Special Issue includes original research articles and reviews on innovative nanocarriers, such as liposomes, metal and carbon nanoparticles, nanocrystals, and polymeric systems, utilizing sustainable and environmentally friendly synthesis methods. Special emphasis is placed on formulation strategies for encapsulating biological macromolecules, advancing the development of efficient, eco-friendly delivery platforms.

## 1. Introduction

Nanotechnology is an interdisciplinary scientific field focused on the design, synthesis, and application of nanostructures and nanomaterials at the nanometer scale [1,2]. The recent surge of interest in this domain has driven the development of nanocarriers for drug delivery applications, including liposomes and their derivatives, metal nanoparticles, carbon-based nanostructures, nanocrystals, polymeric nanospheres, nanocapsules, and micelles [3,4]. These nanocarriers offer promising capabilities to modulate drug release kinetics, enhance the solubility and bioavailability of poorly soluble active pharmaceutical ingredients (APIs), and mitigate systemic side effects [5,6,7]. This Special Issue of Pharmaceutics solicits original research articles and comprehensive reviews that explore innovative applications of nanotechnology within the pharmaceutical field. The focus is on advances in green synthesis methods and the development of novel nanocarriers, particularly those aimed at encapsulating biological macromolecules, thus fostering sustainable and efficient delivery systems for therapeutic agents [8,9].

## 2. Overview of the Published Articles

This Special Issue comprises eleven contributions, including nine original research articles and two comprehensive reviews. The articles by Yoshihara et al. focused on the development and characterization of CPC-montmorillonite-based antimicrobial films for denture coatings. The films demonstrated temperature-dependent, reversible release of CPC and maintained antifungal activity against Candida albicans. These coatings present a promising strategy for preventing denture-associated candidiasis (contribution 1). The articles by Alshaer et al. developed and characterized clarithromycin-loaded bovine serum albumin nanoparticles (CLA-BSA NPs) with optimized properties for enhanced delivery. The CLA-BSA NPs showed strong interaction through van der Waals forces, with a controlled release of over 50% in reductive media. Biological tests demonstrated significant anticancer activity against A549 lung cancer cells, minimal toxicity to healthy fibroblasts, and notable antibacterial effects, especially against *Bacillus cereus*. These findings suggest CLA-BSA NPs are a promising system for improved anticancer and antibacterial therapy (contribution 2). In the paper by Raluca Pauna and colleagues, the anti-inflammatory and antioxidant effects of diclofenac encapsulated in chitosan-coated lipid microvesicles (DCF-m) were evaluated compared to free diclofenac (DCF) in a rat model of subacute inflammation. Briefly, using the cotton pellet granuloma model, rats were divided into four groups (n = 5): negative control, granuloma control, free DCF-treated, and DCF-m-treated. Both DCF and DCF-m significantly reduced granuloma mass, body weight gain, and serum inflammatory markers compared to the granuloma control. Notably, DCF-m exhibited superior anti-inflammatory effects and greater enhancement of antioxidant enzyme activity compared to free DCF. These results suggest that DCF-m possesses enhanced anti-inflammatory and antioxidant efficacy relative to conventional diclofenac in models of subacute inflammation (contribution 3). The research herein by Hawari Mansor et al. developed silk fibroin particles (SFPs) using a novel swirl mixer via microfluidics-assisted desolvation and evaluated their potential for drug delivery. The produced SFPs measured under 200 nm, exhibited uniform size distribution, and remained stable for 30 days. CUR and 5-FU were encapsulated with efficiencies of 37% and 82%, respectively, showing sustained release over 72 h. In vitro, CUR/5-FU-loaded magnetic SFPs induced cytotoxicity and G2/M cell cycle arrest in breast cancer cells while sparing non-cancerous cells, with confirmed cytoplasmic drug uptake. In vivo, magnetic guidance enhanced tumor-specific drug accumulation and increased tumor necrosis. These findings demonstrate that swirl mixer-fabricated bio-inspired SFPs are promising and scalable nanocarriers for targeted breast cancer therapy (contribution 4). This study, conducted by Zapata and colleagues, explores carrier-based strategies to enhance cannabidiol (CBD) bioavailability during digestion. Twenty-four composites incorporating CBD and tailored carbon supports were developed and evaluated under simulated digestive conditions. The antioxidant activity of CBD matched and exceeded that of BHT, with an IC-50 of 10,000 mg/L against SW480 colon carcinoma cells. CBD demonstrated the potential to extend shelf life in lipid and protein foods by 7 and 470 days, respectively. Acid-supported composites showed superior adsorption due to electrostatic interactions, while basic carbons enhanced delivery through electrostatic repulsion. An optimized composite with a CBD loading of 27 mg/g could deliver 1.1 mg, 21.8 mg, and 4 mg to the oral cavity, stomach, and duodenum over 18 h. This work represents a novel approach in designing carriers to improve CBD release and bioavailability in digestive systems (contribution 5). This study by Fischer Karnopp et al. reports the synthesis and characterization of chlorambucil (CLB)-functionalized mesoporous silica nanoparticles (MSNs) sized between 20 and 50 nm to enhance cellular uptake and circulation time. Functionalization was confirmed by FTIR and elemental analysis. Cytotoxicity tests against human lung adenocarcinoma (A549) and colon carcinoma (CT26WT) cells showed that MSN@NH2-CLB exhibited significantly higher cytotoxicity and greater selectivity for cancer cells compared to free CLB. These findings indicate that MSN@NH2-CLB is a promising nanocarrier for targeted cancer therapy (contribution 6). The articles by Bozza et al. explore the impact of neuroinflammation on cerebrovascular and neurological diseases, highlighting impairments in the neurovascular unit and choroid plexus, which can lead to conditions such as idiopathic normal pressure hydrocephalus. Intranasal drug delivery using solid lipid nanoparticles (SLNs), particularly green SLNs derived from natural soaps with antioxidant and anti-inflammatory properties, presents a promising method to bypass the blood–brain barrier. In this study, drug-loaded green SLNs exhibited non-toxicity, reduced cerebrospinal fluid production in vitro, and demonstrated vasoprotective effects along with favorable pharmacokinetics and biodistribution in animal models (contribution 7). Serri et al. investigated the protective effects of hyaluronic acid-based nanoparticles (LicpHA) loaded with Rutin against endothelial damage caused by anthracycline therapies. These nanoparticles were prepared with phosphatidylcholine, cholesterol, poloxamers, and hyaluronic acid using a modified nanoprecipitation technique. Characterization revealed that Rutin influenced the nanoparticles’ size (179 ± 4 nm to 209 ± 4 nm) and surface charge (from −35 ± 1 mV to −30 ± 0.5 mV). Cytotoxicity studies demonstrated that LicpHA Rutin significantly reduced cell death and inflammation compared to epirubicin alone, with lower levels of NLRP3 and other inflammatory markers (*p* < 0.001). The study suggests that Rutin-loaded hyaluronic acid nanoparticles offer significant vasculo-protective effects and warrant further preclinical and clinical investigation for anthracycline-induced vascular damage. (contribution 8). Gambaro et al. optimized lipid nanoparticles (LNPs) for mRNA delivery targeting metabolic diseases by formulating four lipid mixtures (LM1–LM4) with varying components. The LNPs demonstrated stability and homogeneity (75–90 nm), with high mRNA encapsulation efficiency (95–100%). Efficient delivery of EGFP-encoding mRNA to HepG2 and DC2.4 cells was confirmed. Upon exposure to human PBMCs, LNPs induced cytokine secretion, with LM1, LM2, and LM4 eliciting 1.5- to 4-fold increases in IL-8, TNF-α, and MCP-1, while LM3 showed minimal immune activation. Reporter mRNA expression was detected in PBMCs. Hemotoxicity assays indicated good biocompatibility (<2%). In vivo, intramuscular administration in mice resulted in robust mRNA expression primarily in the liver. Modulating LNP lipid components affected reactogenicity, inflammatory responses, and mRNA expression, supporting this platform’s potential for selecting less reactogenic carriers suitable for repeated dosing in metabolic disease therapies (contribution 9). The reviews by Kozak et al. highlight liposome-based targeted drug delivery systems as a major advancement in pharmaceutical science, capable of encapsulating both hydrophilic and hydrophobic drugs. The review discusses how liposomes enhance pharmacokinetics and reduce side effects, particularly in oncology, by enabling precise tumor targeting. It examines various types of liposomes, surface modifications, preparation, and characterization methods, providing an in-depth analysis of their properties and clinical applications. Focusing on active clinical trials and the latest regulatory approvals, the review underscores the transformative potential of liposomal nanocarriers in modern therapy, offering insights into current progress and future innovations (contribution 10). Lipid nanoparticles (LNPs), as discussed in the review by Catenacci et al., trigger immune responses via Toll-like and pattern recognition receptors, causing inflammation and side effects, while adaptive immunity mainly targets the mRNA-encoded protein rather than LNP components. Key factors such as LNP composition, size, shape, surface charge, and protein corona influence immune activation. Understanding these interactions is essential to develop safer, more effective nucleic acid delivery systems. The review also highlights the need for further systematic research to clarify the mechanisms underlying immune responses to LNPs, which will support the design of formulations with improved efficacy and safety (contribution 11).

## 3. Conclusions and Future Directions

Advances in liposomal, lipid–polymer, and hybrid nanocarriers have significantly enhanced stability, targeting specificity, and controlled drug release, showing great potential for treating cancer, neurodegenerative, and metabolic diseases. However, a comprehensive understanding of immune responses and ensuring safety remain significant challenges. Future research should prioritize investigating nanocarrier–immune system interactions, reducing adverse effects, and accelerating clinical translation, with a strong emphasis on sustainable synthesis methods and personalized therapeutic approaches.
